# Assessment of SARS-CoV-2 Genome Sequencing: Quality Criteria and Low-Frequency Variants

**DOI:** 10.1128/JCM.00944-21

**Published:** 2021-09-20

**Authors:** Damien Jacot, Trestan Pillonel, Gilbert Greub, Claire Bertelli

**Affiliations:** a Institute of Microbiology, Laboratory of Genomics and Metagenomics, Lausanne University Hospital and University of Lausanne, Lausanne, Switzerland; University Hospital Munster

**Keywords:** SARS-CoV-2, genome sequencing, variants of concern, quality assessment, contamination, accreditation

## Abstract

Although many laboratories worldwide have developed their sequencing capacities in response to the need for SARS-CoV-2 genome-based surveillance of variants, only a few reported some quality criteria to ensure sequence quality before lineage assignment and submission to public databases. Hence, we aimed here to provide simple quality control criteria for SARS-CoV-2 sequencing to prevent erroneous interpretation of low-quality or contaminated data. We retrospectively investigated 647 SARS-CoV-2 genomes obtained over 10 tiled amplicons sequencing runs. We extracted 26 potentially relevant metrics covering the entire workflow from sample selection to bioinformatics analysis. Based on data distribution, critical values were established for 11 selected metrics to prompt further quality investigations for problematic samples, in particular those with a low viral RNA quantity. Low-frequency variants (<70% of supporting reads) can result from PCR amplification errors, sample cross contaminations, or presence of distinct SARS-CoV2 genomes in the sample sequenced. The number and the prevalence of low-frequency variants can be used as a robust quality criterion to identify possible sequencing errors or contaminations. Overall, we propose 11 metrics with fixed cutoff values as a simple tool to evaluate the quality of SARS-CoV-2 genomes, among which are cycle thresholds, mean depth, proportion of genome covered at least 10×, and the number of low-frequency variants combined with mutation prevalence data.

## INTRODUCTION

The epidemics of COVID-19 (coronavirus disease 2019) caused by SARS-CoV-2 (severe acute respiratory syndrome coronavirus 2) reported in Wuhan City (China) in December 2019 rapidly spread to other regions in China and then to all continents, causing the 2020/2021 pandemic. In December 2020, southeastern England experienced a surge of new SARS-CoV-2 infections with the identification of the first variant of concern (VoC), named B.1.1.7 (1, 2). This first variant was rapidly followed by distinct variants first identified in South Africa (B.1.351) (3), in Brazil (P.1 and P.2) (4, 5), and in India (B.1.617.1/2/3).

The rapid dissemination of these variants elicited concerns over a deterioration of the fragile epidemiological situation and an increased number of infections, adding significant pressure on the health care system. In this context, the European Centre for Disease Prevention and Control (1) and the Swiss government (6) requested the implementation of prescreening strategies (e.g., N501Y and E484K PCRs) to rapidly identify these VoCs and isolate positive cases and their contacts. This triggered the development of SARS-CoV-2 sequencing capacities in numerous diagnostic and research laboratories worldwide to unambiguously assign lineages. The continued infections, in the context of the vaccination campaign, also require the establishment of surveillance programs to identify variants that could escape immunity, causing postvaccinal infections and reinfections.

The expected increase of SARS-CoV-2 genomic data, the potential impact of these results on patient management and public health measures, and the many actors entering the SARS-CoV-2 sequencing business, demand a systematic quality control assessment of these genomes. So far, SARS-CoV-2 genome sequencing has been largely used for epidemiological purposes (7–9) or associated with investigations of nosocomial transmission chains (10). However, most studies, with few exceptions, do not clearly define the quality control criteria used to include or exclude genomic data. Kubik et al. (11) recommended the use of a minimum of 1,000 genome copies per reaction, at least 270,000 reads, and a coverage of >98%, with >75% aligned viral reads for an optimal sequencing process. Similarly, Popa et al. (8) proposed a >96% genome coverage, >80% aligned viral reads, and ≤1,500 uncalled nucleotides. Finally, others proposed an average sequence depth of >200 while using only clinical samples with a reverse transcription-PCR (RT-PCR) cycle threshold (*C_T_*) value below 30 cycles (9).

To implement SARS-CoV-2 genomics in a diagnostic laboratory, where quality criteria are of major importance, we assessed quality metrics extracted from a typical SARS-CoV-2 Illumina sequencing pipeline. These metrics covered specimen selection, nucleic acid extraction, library preparation, and bioinformatics analyses. We propose here critical thresholds on some relevant metrics with a special emphasis on the significance of low-frequency variants. These guidelines should help other laboratories establish simple internal quality controls for SARS-CoV-2 sequencing to avoid interpretation of low-quality or contaminated sample data and to improve the quality of sequences made publicly available.

## MATERIAL AND METHODS

### Library preparation.

RNA from clinical samples (nasopharyngeal or mouth swabs collected in COPAN UTM liquid, 3.5 ml) were extracted using our automated molecular diagnostic platform with a MagNA Pure 96 instrument (Roche, Basel, Switzerland). All samples were processed with the CleanPlex SARS-CoV-2 15 panel and CleanPlex dual indexed (Paragon Genomics number 918011 [12]) according to the manufacturer’s protocol. The CleanPlex SARS-CoV-2 tiled amplicon protocol is made of 343 amplicons distributed into two pools, with amplicon size ranging from 116 to 196 bp (median, 149 bp). No fragmentation is performed (13). PCR products were analyzed using a Fragment Analyzer standard-sensitivity NGS (DNF-473; AATI), and DNA was quantified with a Qubit standard-sensitivity double-stranded DNA (dsDNA) kit (Q32853; Invitrogen). All samples were sequenced using 150-bp paired-end reads on a MiSeq (Illumina, San Diego, CA).

### SARS-CoV-2 sequencing pipeline and validation.

Illumina reads were processed using GENCOV (https://github.com/metagenlab/GENCOV), a modified version of CoVpipe (https://gitlab.com/RKIBioinformaticsPipelines/ncov_minipipe). Briefly, reads were filtered with fastp (14) and mapped on SARS-CoV-2 reference genome NC_045512.2 with bwa (15). The alignment was evaluated with Qualimap (16), and primer sequences from the CleanPlex panel were trimmed with fgbio. Variant calling was performed with Freebayes (parameters: –min-alternate-fraction 0.1 –min-coverage 10 –min-alternate-count 9) (16). Positions covered by fewer than 10 reads were set to N (unknown) if they were not identified as part of a short deletion by Freebayes. Putative variants were filtered with bcftools (17) based on mean mapping quality (MQM; >40) and variant quality (QUAL; >10). Only variants supported by at least 70% of mapped reads were considered to build the consensus of sequenced genomes. The consensus sequence was generated with bcftools and assigned to SARS-Cov-2 lineages with pangolin (2). A diagnostic of consensus sequences was performed with the Nextstrain workflow version 2.0.0.post1 (18). MultiQC (19) was used to aggregate results from the various analyses. To investigate mutations supported by less than 70% of mapped reads (termed “low-frequency variants”), all mutations supported by at least 10% of mapped reads were extracted from Freebayes results. We finally computed the prevalence (presence/absence) of the 19,270 mutations identified in the 647 genomes. The workflow with detailed tool versions can be found on the MetaGenLab github page (https://github.com/metagenlab/GENCOV/releases/tag/1.0). The frequency of individual mutations was calculated in this study based on all samples from 10 sequencing runs. To monitor the quality of the workflow, an internal control was included in the runs. The sequences always passed quality controls and were assigned to the correct lineage (B.1.258.17). The same 30 mutations were identified in all 5 controls. One sample exhibited an additional mutation that was only supported by 10 reads.

### Data availability.

Sequencing reads were submitted to the International Nucleotide Sequence Database Collaboration (INSDC) with project number PRJEB43828. GISAID and ENA accession numbers of raw reads along with the raw data of the metrics are reported in Table S1 in the supplemental material. The bioinformatics workflow is available on GitHub at https://github.com/metagenlab/GENCOV.

## RESULTS

While SARS-CoV-2 genome sequencing and analysis are performed in many laboratories worldwide for epidemiological purposes, no clear criteria have been proposed to assess the quality of SARS-CoV-2 sequences. Critical thresholds or control limits (17, 18) are widely used in clinical laboratories to monitor automated analyses longitudinally. This system could be used in the setting of SARS-CoV-2 genomics to identify abnormal values that may lead to the rejection of sample analysis. To develop critical thresholds for the assessment of SARS-CoV-2 genome quality, data from 647 samples sequenced over 10 sequencing runs were investigated retrospectively.

### SARS-CoV-2 genomics workflow assessment.

**FIG 1:**
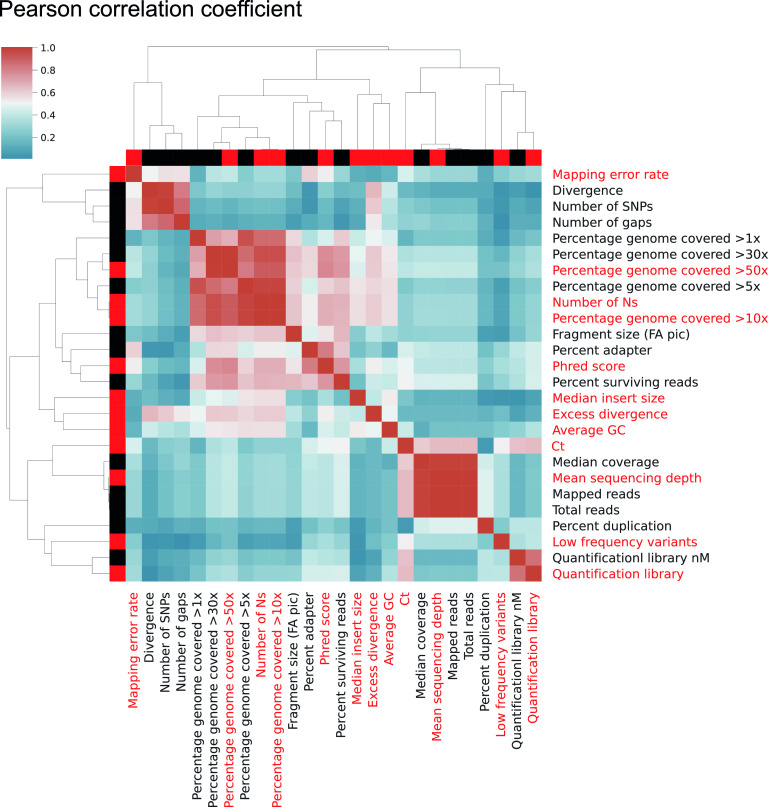
Correlation matrix of the 26 metrics collected from our sequencing workflow. A rational selection of nonredundant metrics was performed to select 11 representative metrics that cover all distinct aspects of the sequencing workflow (red labels).

**FIG 2:**
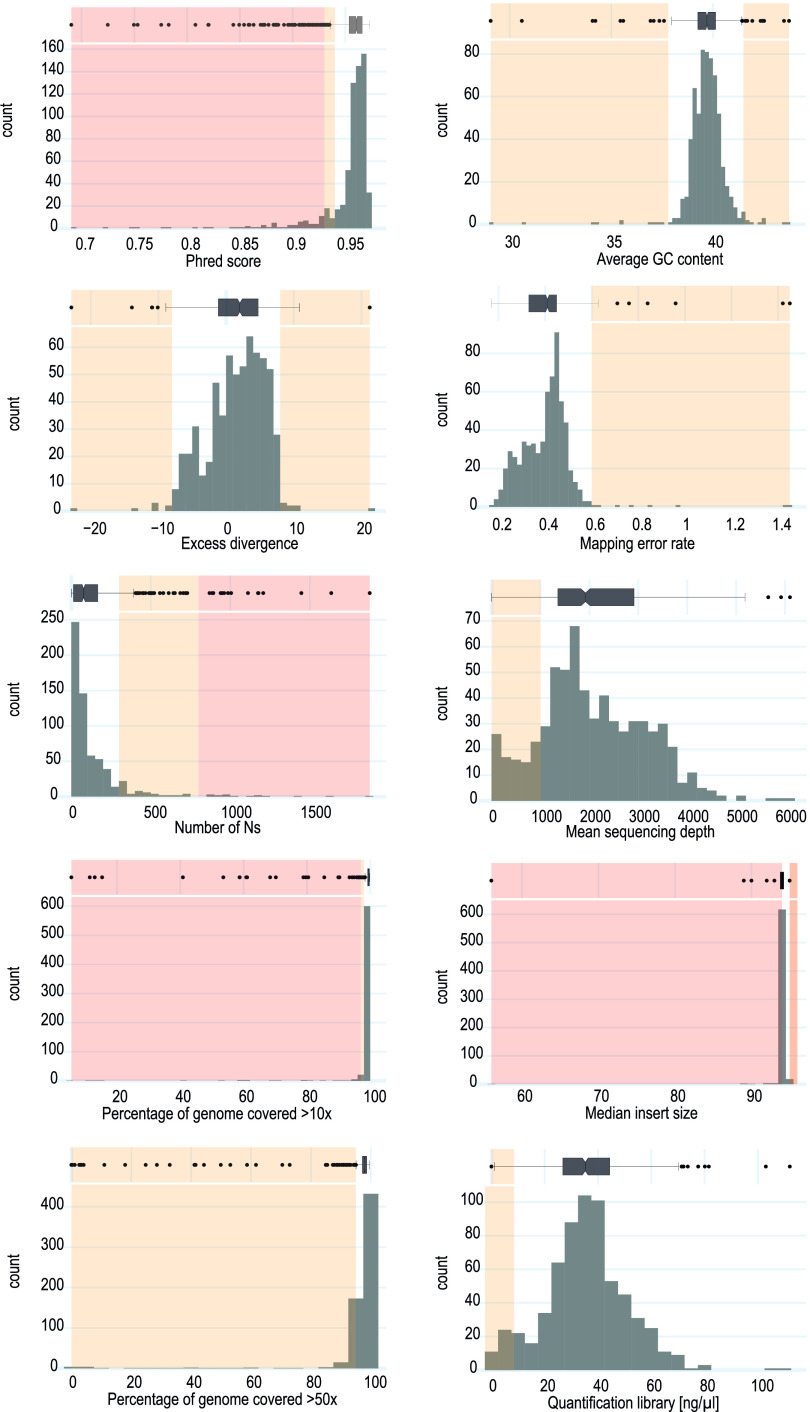
Distribution of the metrics to generate threshold limits. Depending on the expected limit (upper limit, lower limit, or both), all outliers were sequentially and individually investigated to estimate a control limit value.

Twenty-six metrics (see Table S2 in the supplemental material) were collected for their potential to evaluate the quality of SARS-CoV-2 genome sequencing, from DNA extraction to bioinformatics analyses. To reduce redundancy while retaining the ability to evidence problems along the entire workflow, 11 metrics were selected based on expert knowledge in genomics and excluding redundant metrics (Table 1 and Fig. 1). Although nonredundant, some metrics, such as percent duplication or percent surviving rate, were not retained, as they poorly predicted sequencing quality. DNA quantification, library quantification, median insert size measured from mapped read pairs, and read quality (Phred score) allow us to identify eventual problems during library preparation (Fig. 2). The position of the main peak measured by fragment analyzer during library preparation was not included in the 11 quality metrics but remained checked during library amplification. The proportion of genome length covered (>10× and >50×), number of undetermined bases in the consensus genome, average GC content, mean sequencing depth (Fig. 2), and number of variants supported by only 10 to 70% of mapped reads (see below) can be used to assess read quality and genome assembly. A measure of divergence calculated from the expected number of mutations according to the sampling date and a theoretical mutation rate of 25 single-nucleotide polymorphisms (SNP) per year (19) allowed us to detect abnormal sequences with excess or lack of divergence (Fig. 2). However, this threshold limit is dynamic and must be continuously adapted to particular lineages. Indeed, several variants, including the highly contagious B.1.1.7 variant, showed an increased mutation rate (2).

**TABLE 1:** Quality criteria^*a*^

Metric	Median	LCL failed	UCL failed	LCL	UCL
Avg GC	39.69			37.8	41.5
*C_T_*			30		
Excess divergence	1.33			−8	8
Low-frequency variants	0				3
Mapping error rate	0.39				0.6
Mean sequencing depth	2,443.4			1,000	
Median insert size	94	94	96	94	95
No. of Ns	54		800		300
Percent genome covered >10×	99.61	97		98	
Percent genome covered >50×	98.28			95	
q30 rate	0.96	0.93		0.94	
Quantification library (ng/μl)	37.45			8.5	

a Upper and lower control limits (UCL and LCL) were used as critical thresholds to assess the quality of the sequencing. A warning is triggered when a value crosses the corresponding threshold, and the sample is further analyzed using all other metrics. For some metrics, criteria were established to flag likely failed sequencing (UCL failed and LCL failed).

Upper and lower control limits were established based on the distribution of the 11 selected metrics (Fig. 2), with tailored adaptation to account for variable specificities (Table 1). For example, a low read depth might be problematic, whereas a high read depth is, of course, acceptable. Once established, these limits can be used to monitor new sequencing runs and to identify and reject out-of-control values (Fig. S1 and S2). Overall quality for each sample can be scored as the sum of metrics outside control limits.

Clinical specimens contain a diverse range of viral load, from 10^3^ up to 10^9^ copies/ml (20). As clinical samples are often screened by RT-PCRs before SARS-CoV-2 genome sequencing, resulting *C_T_*s can easily be used as a preanalytical criterion. Samples with RT-PCR cycle thresholds above 28 generally resulted in a low number of reads (Fig. S3A), significantly affecting many of the selected quality metrics (Fig. S3B), and the sequences were rejected in the initial manual validation of the samples (Fig. S3C). This cutoff optimized the sequencing success in our setting but should be adapted to other workflow specificities, according to study questions and sample characteristics. Although limited by the number of mouth swabs (*n* = 31), we observed that nasopharyngeal swabs (*n* = 586) resulted in an overall better sequencing quality, likely linked to lower viral loads in mouth swabs or to the presence of PCR inhibitors in saliva (Fig. S3D).

### Low-frequency variants: from amplification errors to contaminations.

Key to the reporting of accurate results is the detection of potential contaminations that could hamper variant calling, affecting cluster analyses and leading to inaccurate lineage definition. The presence of low-frequency variants, defined as positions with variable nucleotides in 10% to 70% of mapped reads (11), could reflect such contaminations between samples. However, this can also be observed due to intrahost heterogeneity (21), uncommon coinfection of different viral strains (22), or PCR polymerase errors (23). However, with an estimated 25 SNP per year (19), corresponding to around two mutations per month or one mutation every two transmissions, multiple variants are unlikely to be found within an individual. Furthermore, such mutations arising randomly in the 29,903 bp of the SARS-CoV-2 reference genome should be observed only rarely, if at all, in other genomes sequenced in the same batch.

**FIG 3:**
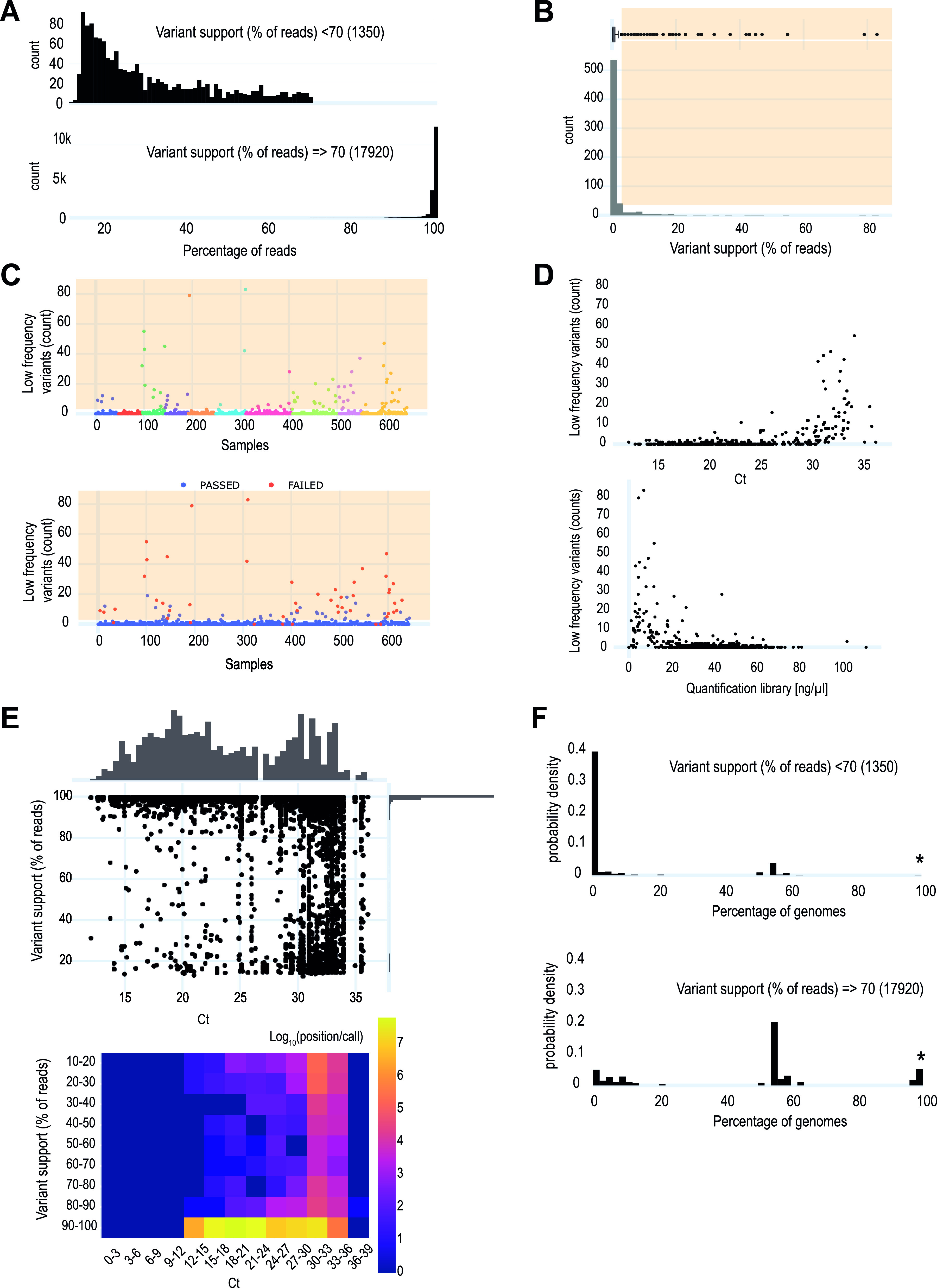
Sequence variants. (A) Distribution of the number of reads supporting a variant. Most variants were supported by >95% of the reads. Low frequency variants (between 10% and 70% of supporting reads) still accounted for 7% of all polymorphisms (1,350/19,270) and showed a dispersed distribution. (B) Counts of low-frequency variants per sample. Most of the samples contained 0 or 2 mixed positions supported by <70% of mapped reads. The orange area indicates defined cutoffs. Values within this area trigger a warning and require further investigations. (C) Distribution of low-frequency variants across the different runs and according to the initial manual validation of the samples. (D) An abrupt accumulation of low-frequency variant calls is observed in specimens with *C_T_*s above 30 or with a low library DNA quantification. (E) Low-*C_T_* samples showed mostly variants supported by 90 to 100% of the reads, whereas high-*C_T_* samples showed an increased heterogeneity with the accumulation of lower frequency variants. (F) Distribution of low-frequency variant calls below (left) and above (right) 70%. Rare mutations (present in <5% of sequenced genomes) are overrepresented in the low-frequency calls (<70%) compared to the position supported by more than 70% of the reads. Stars represent the D614G mutation present in all genomes, whereas the positions found in 50 to 60% of the genomes correspond to B.1.1.7 genomes, overrepresented in our data set.

In our data set, most variants were supported by >95% of the reads (Fig. 3A, right), but low-frequency variants still accounted for 7% of all polymorphisms (1,350/19,270) (Fig. 3A, left). While 68% of samples had no low-frequency variants, a few samples exhibited up to 83 polymorphic sites (Fig. 3B and C). The prevalence of low-frequency variants increased in samples with low nucleic acid concentration (high *C_T_*) or with a low library quantification (Fig. 3D and E), suggesting that these low-frequency variants result from a technical bias. Most low-frequency variants (917/1,350; 67.93%) were unique to a single sample, as opposed to only 2.34% (419/17,920) for highly supported variants (Fig. 3F). These unique low-frequency variants likely result from errors during PCR amplification that occur randomly in the SARS-CoV-2 genome and should only rarely be observed in other genomes, as they do not reflect circulating strains (12). However, low-frequency variants present in other genomes of the sequencing run (Fig. 3F) could represent cross-contaminations (24–26).

For routine analyses, the intrarun prevalence of every variant, including those with low frequency, should be calculated among sequenced samples to distinguish potentially contaminated samples from random incorrect base incorporation during genome amplification. In our setting, if more than three low-frequency variant calls are found in one sample, a manual investigation of the prevalence of these mutations among the other samples of the run is performed. Most samples with several low-frequency variants failed the quality control due to multiple metrics (Fig. S3E). However, some samples harbored a large number of unique low-frequency variants (Table S3), likely as a result of PCR amplification errors. In such cases, the consensus genome sequence can still be used for lineage assignment. Finally, the occurrence of multiple low-frequency variants observed in other samples of the sequencing run is suggestive of a possible cross contamination. This highlights the importance of carefully validating all sequencing metrics. This quality analysis was critical in the following two cases to identify cross-contaminations.

Case 1 included a first nasopharyngeal swab (called sample A) with a *C_T_* of 32, identified as B.1.1.7 lineage by genomic analyses. However, it did not show the characteristic S gene dropout signature of this variant by RT-PCR (27) and presented only 10 of the 17 typical mutations present in B.1.1.7 lineage (Table S4). In the second case, we observed an inconsistency between two samples taken concomitantly from the same patient that resulted in two different lineages. One sample was identified as B.1.1.7 (bucal swab, *C_T_* of 34; named sample B) and the other sample as B.1.160 (nasopharyngeal swab, *C_T_* of 24; named sample C). A close examination of the three sequences showed the presence of several low-frequency variants in samples A (Table S4) and B (Table S5) (46 and 14, respectively) but not in C (Table S6). Further analyses of the prevalence of low-frequency variant calls showed that 20 positions and 9 positions for cases A and B, respectively, were observed in multiple other genomes, which suggested contamination. Low-frequency variants were used here to prevent erroneous lineage assignment. To provide more empirical evidence, we have included additional relevant examples that were identified in samples posterior to this analysis (Table S7).

## DISCUSSION

As previously reported (11, 28), variant calling accuracy and sequencing quality strongly depend on the input material, but very few papers explicitly reported their quality control procedures. As proposed by Kubik et al. (11) and supported by the present retrospective analyses of clinical samples, a *C_T_* of 30 can be used as a preanalytical criterion to exclude samples, but depending on the scientific question, higher *C_T_* could be investigated and quality rigorously assessed. Nasopharyngeal swabs might be preferred to mouth swabs (see Fig. S3D in the supplemental material), and lower respiratory samples can be investigated as prolonged RT-PCR detection of viral RNA in lower respiratory specimens was documented (20, 29). *C_T_* values depend on the reagents and instrument used and, therefore, could vary among laboratories. In our setting, *C_T_*s of 30 correspond to approximately 8.6 × 10^4^ copies/ml of SARS-CoV-2 in the viral transport medium.

Sequencing runs can be monitored using the proposed thresholds (Table 1) in a model of control charts (18) to rapidly identify and investigate problematic sequences (Fig. S2 and S3). The thresholds proposed here are more stringent than those proposed by others (11, 30) but must be used as a warning signal to trigger further analyses and verifications and not as absolute criteria. All metrics must be considered together to assess SARS-CoV-2 genome quality. Indeed, even a moderate mean coverage of the genome could be accepted if the quality of the sequencing data is good, whereas a combination of low coverage and low quality should lead to result rejection.

Only the amplicon-based CleanPlex SARS-CoV-2 kit was evaluated in the present analysis, but the approach can be generalized to other protocols, such as ARTIC (31) or AmpliSeq (32). Some metrics, such as the GC content, the excess divergence, the number of Ns, and the proportion of low-frequency variants, are directly generalizable. For other metrics, we provided here a simple methodology based on value distribution that can be used to adapt thresholds to protocol specificities, particularly for library preparation.

We observed an abrupt increase of low-frequency variants in samples with cycle thresholds above 28. In contrast, lower *C_T_*s showed no or not more than two low-frequency calls. This strongly supports the important effect of technical factors on the accuracy of variant calls, in accordance with independent preliminary findings suggesting that low-copy-number inputs impacted the allele frequency calls and introduced false intrahost mutations (11, 12). In addition, other authors suggested that a significant number of new mutations reported in only one genome submission are likely the result of contamination or recurrent sequencing errors rather than selection or recombination (30, 33). By analyzing the presence and the prevalence of low-frequency variants within sequencing runs or within the whole SARS-CoV-2 population, it is possible to rapidly highlight samples strongly affected by these problems.

In conclusion, we retrospectively analyzed 647 samples over 10 SARS-CoV-2 sequencing runs and identified metrics associated with sequencing quality useful to easily implement quality enhancement measures. To ensure the accuracy of SARS-CoV-2 genome sequences, it is essential to identify and distinguish PCR errors as well as potential sample cross-contaminations. By combining distinct SARS-CoV-2 genomes in one sample, cross contaminations are expected to yield a number of variants detected at lower frequency due to the imbalanced presence of different alleles in the PCR. These low-frequency variants should be shared among the originating contaminating sample and the recipient sample(s). On the other hand, PCR errors are expected to arise randomly throughout the genome and should be unique to each sample. Therefore, we strongly recommend the evaluation of the proportion of low-frequency variants, their prevalence in other SARS-Cov-2 samples from the same sequencing run, and their prevalence in all SARS-CoV-2 sequenced to date as a technical control before data submission to public health agencies and public databases.
