# Supplementary material for: Assessment of SARS-CoV-2 Genome Sequencing: Quality Criteria and Low-Frequency Variants
Source: J Clin Microbiol. 2021 Sep 20;59(10):e00944-21. doi: 10.1128/JCM.00944-21 (PMC8451431; doi:10.1128/JCM.00944-21)
Supplement: Supplemental file 1 — Fig. S1 to S3, Table S1 caption, and Tables S2 to S7. Download JCM.00944-21-s0001.pdf, PDF file, 3.5 MB [file jcm.00944-21-s0001.pdf]

Assessment of SARS-CoV-2 genome sequencing: quality criteria and low frequency variants

Damien Jacot*[1], Trestan Pillonel*[1], Gilbert Greub[1], Claire Bertelli[1]

[1]Institute of Microbiology, Laboratory of genomics and metagenomics, Lausanne University Hospital and University of Lausanne, Lausanne, Switzerland

Supplementary materials

Figure S1

Control chart of the different metrics used as quality controls. Values in the orange areas are spotted as warning and require further investigations whereas value in red areas suggest a failed sequencing run. No constraints were initially applied on the cycle thresholds. The 10 runs are highlighted with different colors.

Figure S2

Control chart of the different metrics used as quality controls. Values in the orange areas are spotted as warning and require further investigations whereas value in red areas suggest a failed sequencing run. No constraints were applied on the cycle thresholds. Sample sequencing was assigned to passed or failed based on an initial manual validation.

Figure S3

A. As expected, low viral loads (high cycle threshold [Ct] values) correlated with a lower number of reads. **B.** Association between the score (scored as the sum of data outside the control limits per sample) and the Ct. The highest quality of sequencing was observed below 27 cycles, samples between 27 and 30 cycles presented intermediate quality, and samples with Cts above 30 were frequently problematic. **C.** Distribution of sample sequencing accepted or failed, according to the SARS-CoV-2 qPCR cycle threshold. **D.** Overall, the sequencing of nasopharyngeal swabs yielded genomes of higher quality as compared to mouth swabs (left). This might be related, at least in part, to the higher Ct values of such specimens (right). **E.** Association between the score and the number of observed low frequency variants.

Figure S1

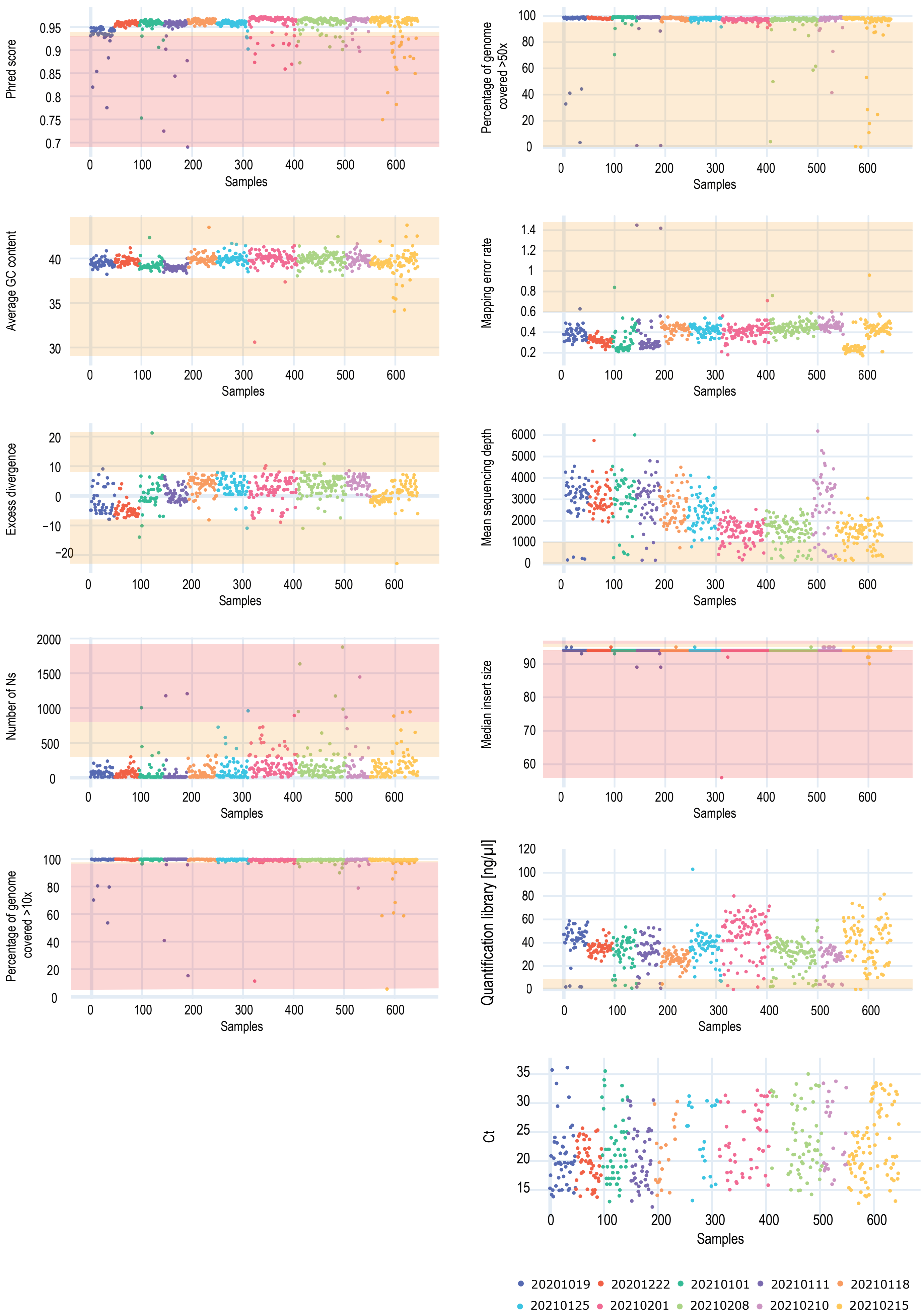

Phred score
Samples
Percentage of genome covered >50x
Samples
Average GC content
Samples
Mapping error rate
Samples
Excess divergence
Samples
Mean sequencing depth
Samples
Number of Ns
Samples
Median insert size
Samples
Percentage of genome covered >10x
Samples
Quantification library [ng/µl]
Samples
Ct
Samples
20201019
20201222
20210101
20210111
20210118
20210125
20210201
20210208
20210210
20210215

Figure S2

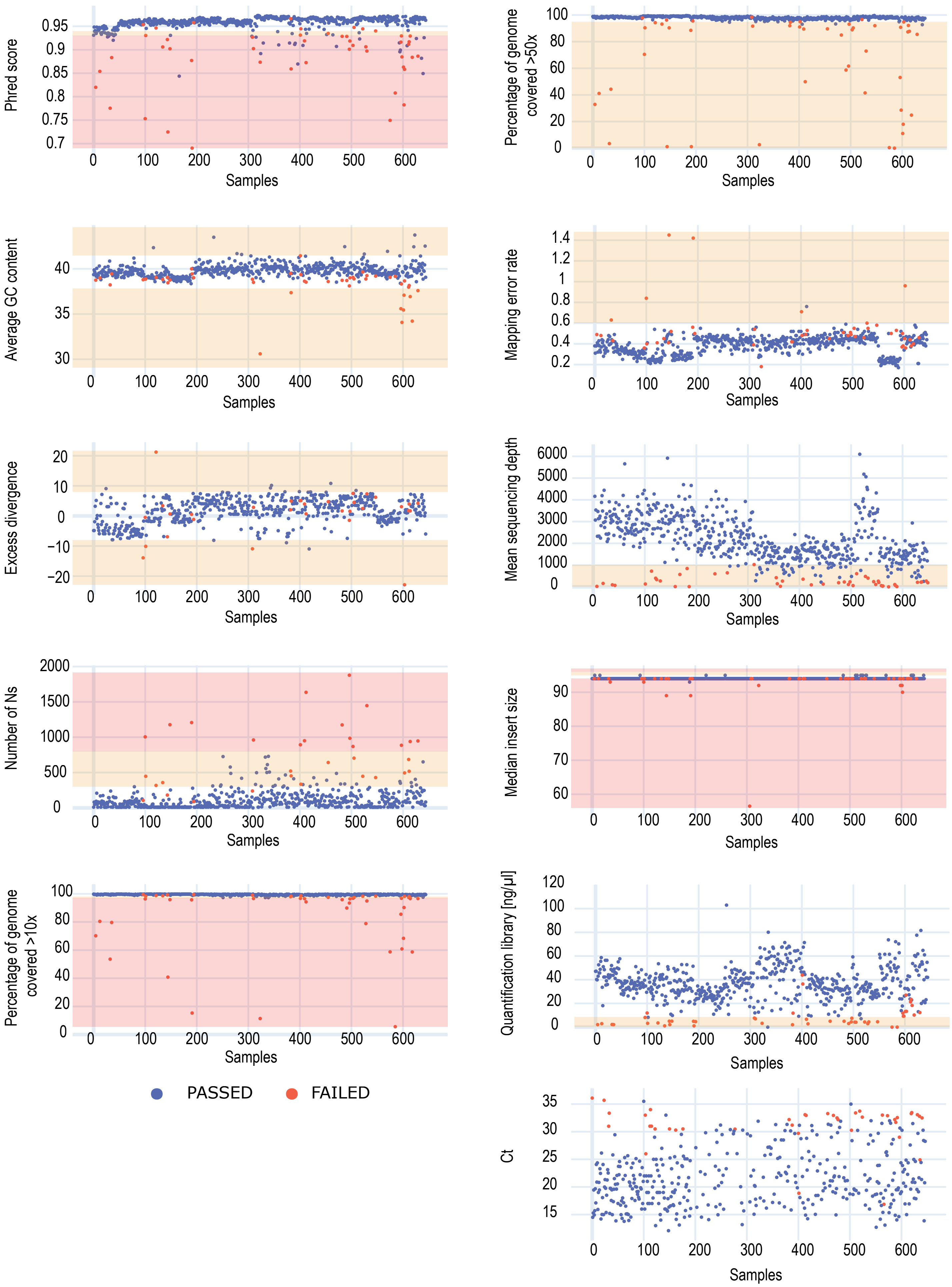

Phred score
Percentage of genome covered >50x
Average GC content
Mapping error rate
Excess divergence
Mean sequencing depth
Number of Ns
Median insert size
Percentage of genome covered >10x
Quantification library [ng/µl]
Ct
Samples
PASSED
FAILED

Figure S3

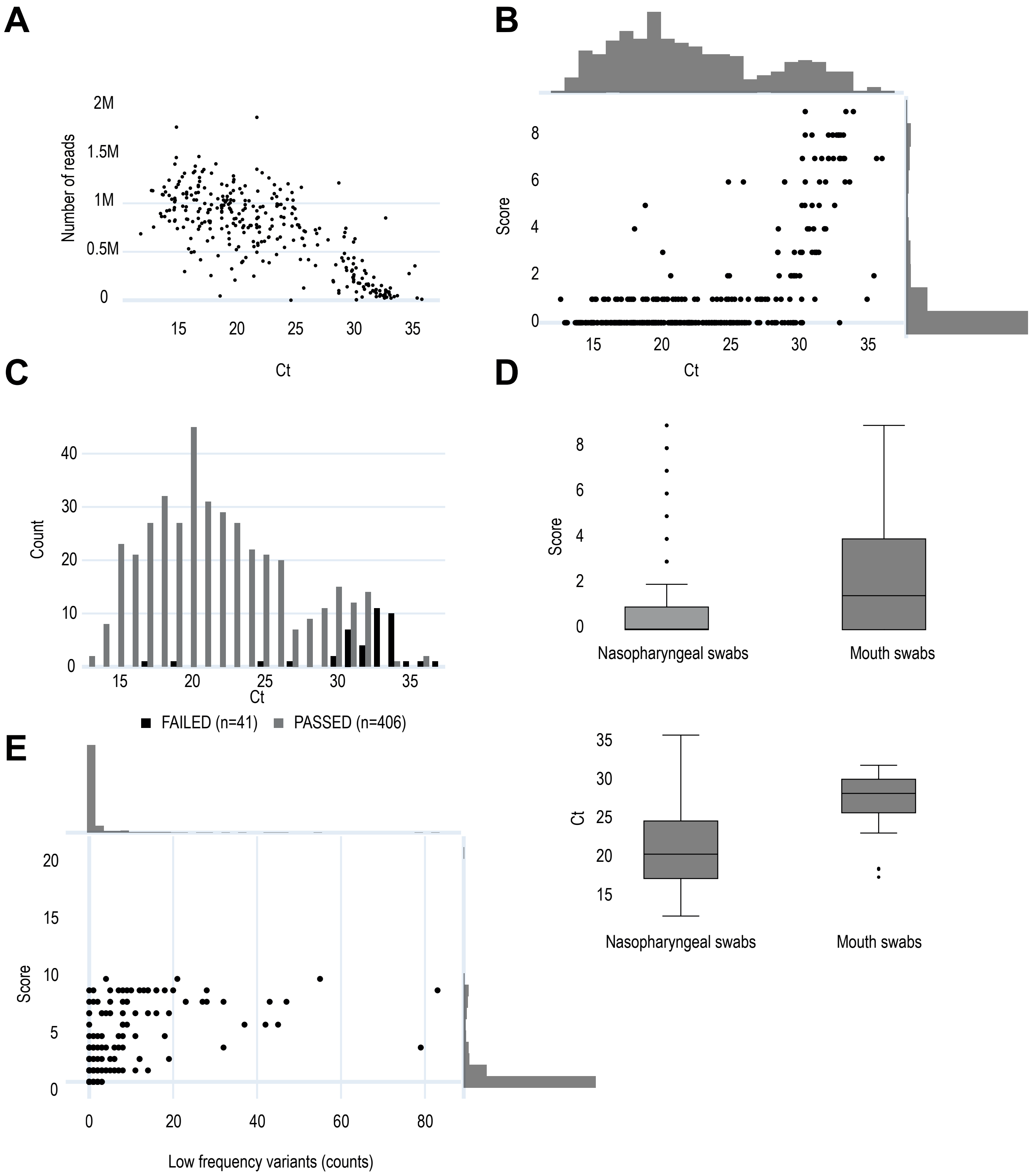
A
2M
1.5M
1M
0.5M
0
Number of reads
15
20
25
30
35
Ct
B
8
6
4
2
0
Score
15
20
25
30
35
Ct
C
40
30
20
10
0
Count
15
20
25
30
35
Ct
FAILED (n=41)
PASSED (n=406)
D
8
6
4
2
0
Score
Nasopharyngeal swabs
Mouth swabs
35
30
25
20
15
Ct
Nasopharyngeal swabs
Mouth swabs
E
20
15
10
5
0
Score
0
20
40
60
80
Low frequency variants (counts)

Table S1

Metrics value, GISAID and ENA accession numbers of the genome sequences and raw sequencing data presented in this study.

Table S2

Metrics investigated for quality assessment and their corresponding definitions. Data from library preparation and sequencing process were analyzed. Metrics highlighted in blue were used for quality measures in our SARS-CoV-2 sequencing pipeline. The name of the bioinformatics tool used for calculation of the metric is described. Cycle thresholds (Ct) were used along with the quantification of the library preparation to assess the amount of starting material needed for sequencing.

**Library preparation**	
Ct	Cycle threshold of the specimen if previously analyzed by RT-PCR
Fragment analyzer size (FA size)	Position of the main pic measured with a fragment analyzer (FA) during library preparation. For the kit used (Paragon Genomics #918011), the pic is found around 270bp
Quantification library	quantification of DNA after library preparation by Qubit in ng/ul
**Sequencing data**	
Adapter	Fastp, percent of adapter sequenced trimmed
Aligned reads	Qualimap, percent of aligned reads
Average GC	Qualimap, average GC content
Divergence	Nextstrain, measure the divergence of the sequenced genome compared to the reference strain. This divergence is proportional to mutation rate of the virus over time.
Duplication	Fastp, percent of duplicated reads
Excess divergence	Nextstrain, score an excess of divergence related to the metric divergence and number of SNPs
Low frequency variants	Low frequency variant calls (reads between 10% to 70%)
Mapped reads	Qualimap, number of mapped reads
Mapping error rate	Qualimap, general alignment error rate
Mean sequencing depth	Qualimap, mean sequencing depth of the genome
Median coverage	Qualimap, median coverage of the genome
Median insert size	Qualimap, median size of the inserts, the CleanPlex SARS-CoV-2 Panel produces amplicon with a median size range of 95 bp.
Number Ns	Nextstrain, number of N in the consensus sequence compared to the reference strain. N were called when a position was covered by less than 10 reads, except if the position was identified as a short deletion by Freebayes.
Number of gaps	Nextstrain, fills the gaps in non-reference sequences with "N" characters. These modifications forces all sequences into the same coordinate space as the reference sequence.
Number of SNPs	Number of SNPs in the consensus sequence compared to the reference strain.
Percent surviving reads	Fastp, percentage of surviving reads alignable on the reference genome after trimming
Percentage genome covered >10x	Qualimap, percent of the genome covered by at least 10 reads
Percentage genome covered >1x	Qualimap, percent of the genome covered by at least 1 reads
Percentage genome covered >30x	Qualimap, percent of the genome covered by at least 30 reads
Percentage genome covered >50x	Qualimap, percent of the genome covered by at least 50 reads
Percentage genome covered >5x	Qualimap, percent of the genome covered by at least 5 reads
Phred score	Fastp, percent of the reads with a Phred score above 30 (q30 rate)
Total reads	Qualimap, number total of reads

Table S3

Example of a low frequency analysis with a sample containing a large number of low frequency variants unique to the sample that likely represent PCR errors expected to arise randomly throughout the genome. Reference and variant count represent the number of reads corresponding restrictively to the reference sequence or the alternative variant. Red area represents the low frequency variant calls (below 70%). In this study, the frequency of individual mutations (prevalence analysis) was calculated based on all samples from ten sequencing runs. Prevalence was calculated for variant calls supported by >70% of mapped reads. Low frequency variants not found in the prevalence analysis (None) are unique mutations likely arising from sequencing errors.

position	VoC	Nucleotide change	depth	Reference count	Variant count	Variant percent	Prevalence analysis (>70%)
241		C241T	164	0	164	100	633
5388	B.1.1.7	C5388A	153	0	153	100	347
5986		C5986T	465	0	465	100	349
6954	B.1.1.7	T6954C	927	0	927	100	352
14408		C14408T	68	0	68	100	630
23063	B.1.1.7 B.1.351 P1	A23063T	207	0	207	100	360
23271	B.1.1.7	C23271A	174	0	174	100	346
23403		A23403G	464	0	464	100	635
24914	B.1.1.7	G24914C	479	0	479	100	352
27643		C27643T	127	0	127	100	49
28111	B.1.1.7	A28111G	895	0	895	100	349
28280	B.1.1.7	G28280C	1591	0	1590	99.94	347
21764	B.1.1.7	ATACATG21764A	844	0	843	99.88	407
23604	B.1.1.7	C23604A	714	1	713	99.86	348
28281	B.1.1.7	A28281T	1592	0	1589	99.81	348
28881		G28881A	1043	2	1041	99.81	372
28883		G28883C	1043	0	1041	99.81	372
16176		T16176C	739	2	737	99.73	349
28270		TA28270T	1592	0	1587	99.69	347
19320		C19320T	282	1	281	99.65	45
28977	B.1.1.7	C28977T	1378	4	1373	99.64	351
27972	B.1.1.7	C27972T	2058	9	2049	99.56	350
28282	B.1.1.7	T28282A	1595	3	1588	99.56	348
28882		G28882A	1043	4	1038	99.52	372
10376		C10376T	165	1	164	99.39	56
913		C913T	309	2	307	99.35	350
21990	B.1.1.7	TTTA21990T	758	0	753	99.34	355
15279		C15279T	229	2	227	99.13	349
28396		G28396A	2775	24	2747	98.99	47

11287	B.1.1.7	GTCTGGTTT T11287G	588	0	582	98.98	358
24506	B.1.1.7	T24506G	96	0	95	98.96	351
23709	B.1.1.7	C23709T	180	2	178	98.89	346
14676		C14676T	48	1	47	97.92	349
10846		G10846T	687	20	667	97.09	3
3037		C3037T	89	3	86	96.63	632
199		G199T	167	1	161	96.41	49
28048	B.1.1.7	G28048T	51	2	49	96.08	303
29669		A29669T	83	4	79	95.18	1
3267	B.1.1.7	C3267T	66	4	62	93.94	346
14316		G14316A	170	32	138	81.18	3
28320		C28320T	3374	1141	2230	66.09	None
27225		G27225T	148	64	84	56.76	None
875		CT875G	66	35	31	46.97	None
13836		G13836T	510	302	205	40.2	None
1076		C1076T	221	134	87	39.37	None
24431		C24431T	312	202	110	35.26	None
11299		G11299T	592	390	200	33.78	None
29526		A29526G	642	446	195	30.37	None
8814		C8814T	433	304	129	29.79	None
28006		C28006T	52	37	15	28.85	None
28011		C28011T	52	37	15	28.85	None
16587		TG16587T	541	380	154	28.47	None
22877		A22877C	144	104	40	27.78	None
14919		C14919T	77	57	20	25.97	None
27059		C27059T	129	96	33	25.58	None
10148		C10148T	446	334	112	25.11	None
29544		A29544G	642	479	155	24.14	None
24824		C24824T	128	98	30	23.44	None
16647		G16647T	676	522	154	22.78	None
4158		CTG4158C	112	86	24	21.43	None
24341		T24341C	151	119	32	21.19	None
7125		C7125T	736	586	150	20.38	None
9111		G9111T	564	450	112	19.86	None
16337		T16337C	52	42	10	19.23	None
25177		G25177T	272	221	51	18.75	None
18866		T18866C	1376	1120	256	18.6	None
18818		A18818T	1387	1127	248	17.88	None
21789		C21789T	846	704	142	16.78	None
21793		G21793T	841	700	141	16.77	None
29625		C29625T	1539	1287	252	16.37	None
29641		A29641C	1542	1287	252	16.34	None
27826		T27826C	399	336	63	15.79	None
27830		T27830C	399	336	63	15.79	None
25852		T25852C	686	578	108	15.74	None
5502		GA5502G	546	460	84	15.38	None

17634		CAAA17634<br>C	1497	1269	221	14.76	None
4068		C4068T	266	226	38	14.29	None

Table S4

Low frequency analysis of case 1, sample A. Reference and variant count represent the number of reads corresponding only to the reference sequence or the alternative variant. Red area represents the low frequency variant calls (below 70%). In this study, the frequency of individual mutations (prevalence analysis) was calculated based on all samples from ten sequencing runs. Prevalence was calculated for variant calls supported by >70% of mapped reads. Low frequency variants not found in the prevalence analysis (None) are unique mutations likely arising from sequencing errors.

position	VoC	Nucleotide change	depth	Reference count	Variant count	Variant percent	Prevalence analysis (>70%)
241		C241T	403	0	403	100	633
3037		C3037T	169	0	169	100	632
23063	B.1.1.7 B.1.351 P1	A23063T	131	0	131	100	360
23403		A23403G	322	1	321	99.69	635
14408		C14408T	723	3	718	99.31	630
24506	B.1.1.7	T24506G	173	3	170	98.27	351
11287	B.1.1.7	GTCTGGTTTT 11287G	247	7	240	97.17	358
17615		A17615G	266	8	258	96.99	57
23709	B.1.1.7	C23709T	121	5	116	95.87	346
3267	B.1.1.7	C3267T	97	5	92	94.85	346
28881		G28881A	250	13	237	94.8	372
28882		G28882A	250	12	237	94.8	372
28883		G28883C	250	13	237	94.8	372
14064		T14064C	1795	244	1550	86.35	3
9430		C9430T	1305	251	1053	80.69	9
27972	B.1.1.7	C27972T	880	181	698	79.32	350
21867		G21867T	117	26	91	77.78	5
6954	B.1.1.7	T6954C	398	103	295	74.12	352
6441		A6441G	458	121	337	73.58	3
5986		C5986T	329	93	236	71.73	349
23856		G23856T	408	119	289	70.83	1
9204		A9204G	722	241	481	66.62	3
29445		C29445T	809	294	514	63.54	2
24914	B.1.1.7	G24914C	371	154	217	58.49	352
16176		T16176C	610	254	356	58.36	349
28321		G28321T	1671	705	964	57.69	2
2258		G2258A	432	183	249	57.64	1
23271	B.1.1.7	C23271A	349	153	196	56.16	346
21575		C21575T	513	228	285	55.56	1
913		C913T	204	93	111	54.41	350
3548		GGACCACTT A3548G	456	209	234	51.32	None
2647		A2647G	518	266	252	48.65	None
19672		C19672T	312	171	140	44.87	None

23422		C23422T	321	177	143	44.55	None
23381		C23381T	323	180	143	44.27	None
17821		C17821T	544	310	234	43.01	None
6625		A6625G	591	337	254	42.98	None
13599		T13599C	696	397	298	42.82	None
11497		C11497T	1043	598	445	42.67	27
9355		GT9355G	1312	732	559	42.61	None
24933		G24933T	361	213	147	40.72	1
28282	B.1.1.7	T28282A	953	595	356	37.36	348
28280	B.1.1.7	G28280C	945	596	349	36.93	347
28281	B.1.1.7	A28281T	945	596	349	36.93	348
28270		TA28270T	950	586	347	36.53	347
5388	B.1.1.7	C5388A	395	255	140	35.44	347
20925		G20925T	1726	1118	606	35.11	None
10650		CTATTACAGT 10650C	792	527	261	32.95	None
4319		G4319C	1076	734	340	31.6	None
15766		G15766T	912	625	287	31.47	28
28111	B.1.1.7	A28111G	1445	999	446	30.87	349
14676		C14676T	1457	1025	431	29.58	349
8140		C8140T	782	563	217	27.75	2
23604	B.1.1.7	C23604A	743	541	200	26.92	348
25563		G25563T	1303	990	310	23.79	40
26876		T26876C	1392	1077	315	22.63	28
18652		C18652T	1091	851	240	22	None
21990	B.1.1.7	TTTA21990T	1515	1159	321	21.19	355
19983		CT19983C	2246	1744	458	20.39	None
28679		G28679A	2727	2188	539	19.77	None
12162		A12162G	2122	1747	374	17.62	16
19686		AC19686A	4242	3523	673	15.87	None
29266		G29266T	956	808	148	15.48	None
17334		G17334T	1073	905	166	15.47	None
28977	B.1.1.7	C28977T	2575	2178	394	15.3	351
11074		C11074CT	94	70	14	14.89	None

Table S5

Low frequency analysis of case 2, sample B. Example of a low frequency analysis with a sample containing a large number of low frequency variants uniquely found. Reference and variant count represent the number of reads corresponding restrictively to the reference sequence or the alternative variant. Red area represents the low frequency variant calls (below 70%). In this study, the frequency of individual mutations (prevalence analysis) was calculated based on all samples from ten sequencing runs. Prevalence was calculated for variant calls supported by >70% of mapped reads. Low frequency variants not found in the prevalence analysis (None) are unique mutations likely arising from sequencing errors.

position	VoC	Nucleotide change	depth	Reference count	Variant count	Variant percent	Prevalence analysis (>70%)
241		C241T	83	0	83	100	633
3037		C3037T	141	0	141	100	632
23063	B.1.1.7<br>B.1.351 P1	A23063T	159	0	159	100	360
23271	B.1.1.7	C23271A	144	0	144	100	346
28111	B.1.1.7	A28111G	356	0	356	100	349
14408		C14408T	674	1	673	99.85	630
23403		A23403G	340	0	339	99.71	635
23604	B.1.1.7	C23604A	157	1	156	99.36	348
14676		C14676T	478	4	474	99.16	349
6954	B.1.1.7	T6954C	353	3	350	99.15	352
28280	B.1.1.7	G28280C	339	3	336	99.12	347
28282	B.1.1.7	T28282A	339	3	336	99.12	348
27972	B.1.1.7	C27972T	610	7	603	98.85	350
21990	B.1.1.7	TTTA21990T	430	3	425	98.84	355
28281	B.1.1.7	A28281T	339	4	335	98.82	348
28270		TA28270T	338	2	333	98.52	347
15279		C15279T	167	3	164	98.2	349
12162		A12162G	459	9	450	98.04	16
24506	B.1.1.7	T24506G	187	4	183	97.86	351
913		C913T	179	4	175	97.77	350
28977	B.1.1.7	C28977T	218	5	213	97.71	351
21867		G21867T	117	3	114	97.44	5
5986		C5986T	285	8	277	97.19	349
3267	B.1.1.7	C3267T	55	2	53	96.36	346
11287	B.1.1.7	GTCTGGTTT<br>T11287G	203	8	195	96.06	358
24914	B.1.1.7	G24914C	208	9	199	95.67	352
23709	B.1.1.7	C23709T	135	9	126	93.33	346
5388	B.1.1.7	C5388A	114	12	102	89.47	347
21764	B.1.1.7	ATACATG21<br>764A	21	2	17	80.95	407

9526	G9526T	997	191	806	80.84	29
755	A755G	546	198	348	63.74	None
770	G770A	546	198	348	63.74	None
14412	A14412G	678	260	418	61.65	None
18877	C18877T	753	330	423	56.18	28
26876	T26876C	520	236	284	54.62	28
28883	G28883C	416	196	220	52.88	372
17615	A17615G	493	234	259	52.54	57
28881	G28881A	416	198	218	52.4	372
28882	G28882A	416	200	216	51.92	372
17019	G17019T	392	205	186	47.45	28
16176	T16176C	504	274	230	45.63	349
5629	G5629T	493	271	222	45.03	28
24799	T24799C	46	33	12	26.09	1
15649	G15649A	1480	1121	357	24.12	None

Table S6

Low frequency analysis of case 2, sample C. No low frequency variant was observed and all calls were supported by >90% of the reads. Reference and variant count represent the number of reads corresponding restrictively to the reference sequence or the alternative variant. Red area represents the low frequency variant calls (below 70%). In this study, the frequency of individual mutations (prevalence analysis) was calculated based on all samples from ten sequencing runs. Prevalence was calculated for variant calls supported by >70% of mapped reads. Low frequency variants not found in the prevalence analysis (None) are unique mutations likely arising from sequencing errors.

position	VoC	Nucleotide change	depth	Reference count	Variant count	Variant percent	Prevalence analysis (>70%)
9526		G9526T	2589	2	2585	99.85	1
26735		C26735T	2686	4	2682	99.85	1
23403		A23403G	3088	5	3083	99.84	44
13051		C13051T	1723	3	1720	99.83	1
14408		C14408T	2361	2	2357	99.83	45
26876		T26876C	3086	6	3080	99.81	1
241		C241T	1928	4	1924	99.79	45
25563		G25563T	4917	6	4905	99.76	2
4543		C4543T	4065	8	4055	99.75	1
15766		G15766T	1959	3	1954	99.74	1
22992		G22992A	2685	7	2678	99.74	1
3037		C3037T	1813	4	1808	99.72	45
5629		G5629T	6274	7	6255	99.7	1
24734		C24734T	2311	5	2303	99.65	1
25710		C25710T	1956	4	1949	99.64	1
29557		G29557T	2480	4	2471	99.64	1
27964		C27964T	10139	29	10100	99.62	1
23683		C23683T	2297	7	2288	99.61	1
13993		G13993T	3358	9	3341	99.49	1
11497		C11497T	4067	20	4046	99.48	1
18877		C18877T	5144	19	5117	99.48	1
16889		A16889G	4526	23	4502	99.47	1
29399		G29399A	17781	82	17673	99.39	1
17019		G17019T	4642	7	4613	99.38	1
28975		G28975C	9643	40	9313	96.58	1
27434		C27434T	2483	264	2218	89.33	1

Table S7

Summary table showing five examples of low frequency variant calls containing samples. Cases 1-5 support possible cross-contamination between samples with low frequency variant calls shared across multiple samples of the run. Cases 6-10 support PCR errors expected to arise randomly throughout the genome and unique to each sample.

Case	N. variants supported by <70% of reads	N. unique variants supported by <70% of reads	Mean depth	Interpretation	Comments
1	46	5 (11%)	722.4606	Contaminated	Presence of 17/19 B.1.1.7 defining mutations, 10/17 supported by <70% of the reads. Resequencing resulted in a different lineage (B.1.234) with 23 SNPs compared to the first sequencing attempt. B.1.234 was consistent with partner results
2	14	5 (36%)	271.3217	Contaminated	Identified as B.1.1.7. The original mouth sample was sequenced twice without success. Sequencing of a nasopharyngeal swab identified a different lineage (B.1.160)
3	16	4 (25%)	397.1342	Contaminated	Sample isolated in March 2020 with very high excess divergence, contaminated by B.1.1.7 sequences
4	11	3 (27%)	2061.9582	Contaminated	Sample isolated in March 2020, contaminated by B.1.1.7 sequences
5	55	39 (71%)	130.7428	Contaminated	Identified as B.1.160, but 8/19 B.1.1.7 defining mutations among low frequency variants.
6	28	28 (100%)	352.4755	Non-contaminated	Only unique low frequency variants. Re-sequencing resulted in nearly identical results (37/38 shared mutations)
7	83	82 (82%)	644.9949	Non-contaminated	82% of unique low frequency variants. Re-sequencing gave consistent results (B.1.160.29 lineage, 26/32 shared mutations)
8	22	21 (95%)	4519.2302	Non-contaminated	95% of unique low frequency mutations. Multiple re-sequencing resulted in nearly identical results (41/43 shared mutations)
9	20	20 (100%)	231.9377	Non-contaminated	Only unique low frequency variants. Re-sequencing resulted in nearly identical results (35/38 shared mutations)
10	14	14 (100%)	224.221	Non-contaminated	Only unique low frequency variants. Re-sequencing resulted in nearly identical results (37/38 shared mutations)
